# Tricky Topology: Persistence of Folded Human Telomeric i-Motif DNA at Ambient Temperature and Neutral pH

**DOI:** 10.3389/fchem.2020.00040

**Published:** 2020-01-31

**Authors:** Mahmoud A. S. Abdelhamid, Zoë A. E. Waller

**Affiliations:** ^1^School of Pharmacy, University of East Anglia, Norwich Research Park, Norwich, United Kingdom; ^2^Centre for Molecular and Structural Biochemistry, University of East Anglia, Norwich Research Park, Norwich, United Kingdom

**Keywords:** i-motif, cytosine, quadruplex, DNA, topology, kinetics

## Abstract

i-Motifs are four-stranded DNA structures formed from sequences rich in cytosine, held together by hemi-protonated cytosine-cytosine base pairs. These structures have been utilized extensively as pH-switches in DNA-based nanotechnology. Recently there has been an increasing interest in i-motif structures in biology, fuelled by examples of when these can form under neutral conditions. Herein we describe a cautionary tale regarding handling of i-motif samples. Using CD and UV spectroscopy we show that it is important to be consistent in annealing i-motif DNA samples as at neutral pH, i-motif unfolding kinetics is dependent on the time allowed for annealing and equilibration. We describe how the quadruplex structure formed by the human telomeric i-motif sequence can be shown to form and persist in the same conditions of neutral pH and ambient temperature in which, once at thermodynamic equilibrium, it exists predominantly as a random coil. This study has implications not only for work with i-motif DNA structures, but also in the uses and applications of these in nanotechnological devices.

## Introduction

The i-motif is a quadruplex DNA structure formed from cytosine-rich sequences and has wide-ranging uses in nanotechnology as well as having potential to impact biological processes (Gehring et al., 1993; Alberti et al., 2006; Abou Assi et al., 2018). While sequences have been identified which form i-motif at pH > 7 (Brazier et al., 2012; Wright et al., 2017a; Dzatko et al., 2018), the dependence of i-motif formation on acidic pH is much better understood (Day et al., 2014; Dembska, 2016). This is owing to the structure's cytosine core, consisting of intercalated cytosine-cytosine^+^ base pairs, where the N3 of one of the pair of cytosines is protonated. Other factors such as molecular crowding (Cui et al., 2013; Tateishi-Karimata et al., 2013; Saxena et al., 2017), low temperature (Zhou et al., 2010; Nguyen et al., 2017), silver (I) (Day et al., 2013) and copper (I) (Abdelhamid et al., 2018) cations and negative superhelicity (Sun and Hurley, 2009) have been shown to be favorable for formation of i-motif.

Much work has been done to understand the behavior of i-motif structures in different environments including studying the effect of varying the cytosine tract and loop lengths (Arora et al., 2009; Gurung et al., 2015), using epigenetically modified cytosine analogs (Bhavsar-Jog et al., 2014; Xu et al., 2015), and modification of the DNA backbone (Assi et al., 2016), amongst other modifications (Tsvetkov et al., 2018; Školáková et al., 2019). Owing to the importance of pH in the control of folding, pH has been examined extensively to understand its effect on the i-motif to learn about its stability at different pH values and its effect on the thermodynamic and kinetic properties of the structure. On the other hand, the effect of temperature on the structure has been studied much less comprehensively. Unusual temperature-dependent effects have been observed previously for the i-motif in the context of exploring its response to changes in pH. This includes the hysteresis that is commonly observed between thermal melting and annealing curves for the structure (Mergny and Lacroix, 2003; Wright et al., 2017a), as well as isothermal hysteresis in pH transitions (Rogers et al., 2018). Kinetic partitioning has also been observed, where the i-motif will initially fold rapidly into one conformation in response to a decrease in pH and then, over time, unfold and refold to a slower forming, but more stable conformation (Lieblein et al., 2012). This led to the description of the i-motif structure existing in an equilibrium of slowly interconverting conformers at a given pH and temperature (Lieblein et al., 2013). As a result of this dynamic nature, the effect of temperature is an important variable that should be given due consideration.

The sequence from the human telomeric region 3′-(AATCCC)_n_-5′, and variants with slight modifications, have been used frequently by researchers as a model system for learning about i-motif structure (Phan and Mergny, 2002; Chen et al., 2012; Jonchhe et al., 2018). The i-motif from the human telomeric region has previously been shown to be able to form at pH 7.0 at low temperature (Zhou et al., 2010). To the best of our knowledge, this is the only study to directly examine the effect of low temperature on the i-motif at neutral or slightly alkaline pH values. In their experiments, Zhou et al. showed that dilution of HT (a sequence based on the cytosine-rich human telomeric region) into pH 7.0 buffer pre-incubated at 4°C, led to intramolecular i-motif formation within 700 s. Herein, we describe another peculiar temperature-dependent phenomenon observed during the course of working with the human telomeric i-motif at pH values >7. We found that at pH 7.11 the sequence from the human telomere (5′-(CCC-TAA)_4_-3′, hTeloC) will still form i-motif when cooled to 4°C, albeit more slowly than what was observed by Zhou et al. Most interestingly however, was the observation that extended incubation times at low temperature resulted in a significant change in the unfolding kinetics of the structure, resulting in much slower unfolding at 20°C. This has implications on how samples should be handled before, during and between experiments, to have consistent and comparable observations and prevent unintended convolution of results. Crucially though, as the i-motif is used extensively in nanotechnological applications, this presents another way for researchers to achieve more precise control of the dynamic equilibrium of this exquisite structure.

## Materials and Methods

### Oligonucleotide Synthesis and Sample Preparation

The hTeloC oligonucleotide 5′-[(TAA-CCC)_4_]-3′ was supplied by Eurogentec (Belgium), synthesized on a 200 nmol scale and purified by reverse phase HPLC. hTeloC was dissolved at approximately 1 mM in ultrapure water (18.2 MΩ·cm) and quantified using a nanodrop. DNA Samples for experiments were thermally annealed at 10 μM in 10 mM sodium cacodylate buffer at pH 7.11 in a thermal heating block by holding at 95°C for 5 min followed by slow cooling to room temperature overnight.

### Circular Dichroism (CD) and Ultraviolet (UV) Spectroscopy

CD and UV spectra were recorded on a Jasco J-810 spectropolarimeter equipped with a Peltier thermostatted single cell holder, a PTC-423S temperature controller and a MCB-100 mini circulation bath set to 4.0°C. Each sample volume was 100 μL and was measured using a 1 mm path length quartz cuvette. The equilibrations at 4°C for 3 and 6 h were performed *in situ* in the spectrometer; the temperature of the sample holder was set to 4°C and acquisition of spectra began when the sample holder's internal temperature probe reached the target temperature. For the longer equilibration times samples were stored at 4°C in a refrigerator for the indicated time. Following the 4°C equilibrations the temperature of the sample holder was set to 20°C and acquisition of spectra began when the sample holder's internal temperature probe reached the desired temperature. Spectra were acquired using the Jasco Spectra Manager application's Interval Scan Measurement program with the parameters set to measure from 320 to 200 nm, with a scanning speed of 200 nm/min, response time of 1 s, 0.5 nm data pitch and 2 nm bandwidth. A time interval between 2 and 5 min was used depending on the experiment. Melting experiments were performed using the Temperature/Wavelength Scan program using the same measurement parameters as above while heating the sample at a rate of 1°C/min from 5 to 95°C and measuring at 5°C intervals. In the case of the melting experiments as time was not a variable each data point was able to be acquired via an accumulation of four scans.

### Sample Temperature Monitoring

A HANNA instruments type K thermocouple was used to directly measure the temperature of 100 μL samples in the 1 mm quartz cuvettes when inside the cell holder of the spectropolarimeter to determine the delay between the temperature reported by the sample holder's probe and the temperature of the sample itself. For the cooling from 20 to 4°C this was found to be within 3 min and for the transition from 4 to 20°C this was found to be <1 min.

## Results and Discussion

The sequence hTeloC has a transitional pH of 6.11 at room temperature in 10 mM sodium cacodylate buffer with 100 mM sodium chloride (Wright et al., 2017b). This is the pH at which the population is evenly split between the folded and unfolded states. A lower pH would result in the sequence being predominantly folded into the i-motif and the inverse true at higher pH. Cytosine-cytosine base pair stability is optimum when the pH is equal to the p*K*_a_ of the cytosine, however cytosines can still pick up free H^+^ from solution, even at neutral pH but these cytosine-cytosine base pairs are less stable than they would be at a lower pH (Mergny et al., 1995). The stimulus for this study was the observation that a sample of hTeloC, which had been annealed at 10 μM in 10 mM sodium cacodylate buffer at pH 7.0, appeared to be folding into i-motif over time. Samples had been prepared as described commonly in the literature: the oligonucleotide was diluted to the desired concentration (10 μM) in buffer (10 mM sodium cacodylate) and held in a heating block at 95°C for 5 min and then allowed to cool to room temperature slowly overnight. This is done to maintain the uniformity of the population and allow the adoption of the equilibrium structure under those conditions. Finally, measurements were being collected at room temperature (~20°C).

**Figure 1:**
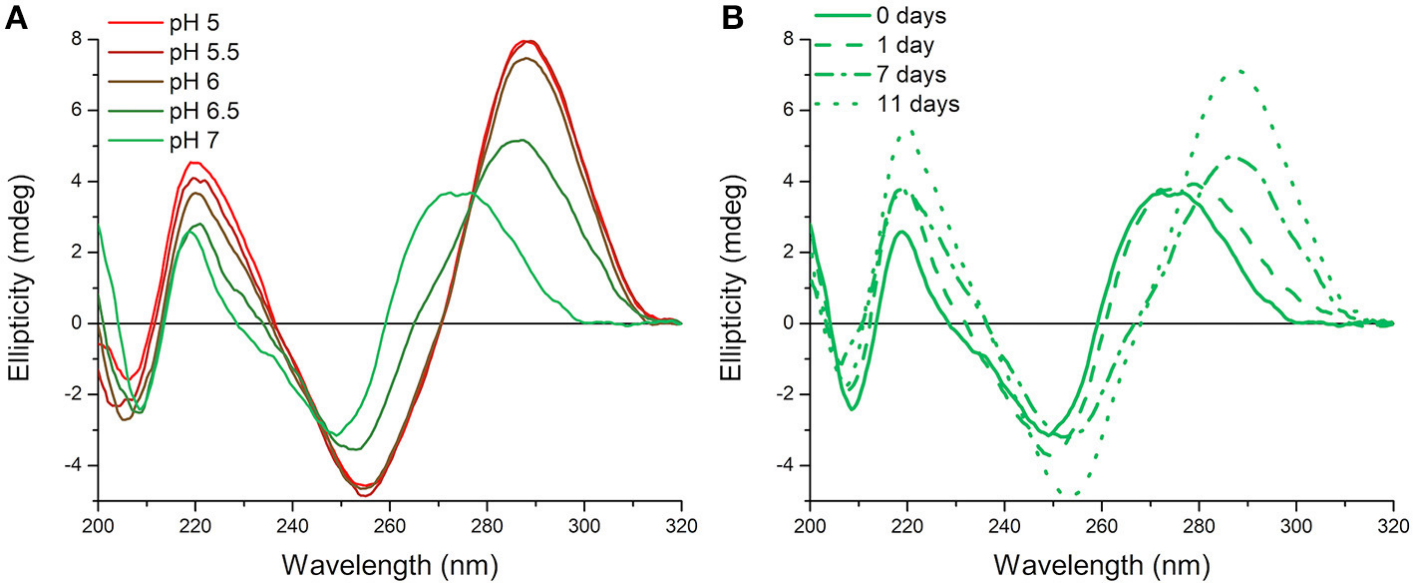
CD spectra of 10 μM hTeloC in 10 mM sodium cacodylate buffer at room temperature. **(A)** Spectra of samples at different pH values measured without storage at low temperature. **(B)** Spectra of samples at pH 7.0 measured after storage at 4°C for the indicated time.

Originally measurements at room temperature and pH 7.0 were used so that the hTeloC sequence would predominantly be unfolded (Figure 1A). However, it became apparent that, over time, this sample appeared to be folded, as evidenced by the characteristic CD spectrum of folded i-motif, similar to that observed at pH 5 (Figure 1B). The only change that was taking place over time was a difference in the temperature: samples were stored in a refrigerator (4°C) between measurements, but enough time was allowed for them to return to room temperature before measurement. Previous work on the human telomeric i-motif had shown that it was possible to fold at neutral and slightly alkaline pH at low temperature, but as the temperature was raised to room temperature, the structure unfolds to a random coil. The melting temperature for the structure at pH 7.0 was 13°C, and above this temperature the structure was previously shown to return to an unfolded state (Zhou et al., 2010). Therefore, it was initially presumed that temporary cold storage would have little effect on measurements taken at room temperature, providing the sample was adequately equilibrated at room temperature.

To better understand what was happening, new samples were prepared in the same way, in this case at pH 7.11, to go above the pH used in the experiments described by Zhou et al. The possibility that under these conditions the i-motif had very slow folding kinetics at room temperature (~20°C) and was forming at pH 7.0 over the course of several days was considered. However, this was determined not to be the case as samples kept at room temperature did not exhibit this folding form over time (Supplementary Figure 1). On the other hand, samples stored at 4°C were found to fold into i-motif over time, shown by the presence of a characteristic i-motif spectrum in the CD (Supplementary Figures 2A,C). To determine the folding kinetics of i-motif formation, samples were monitored as a function of both the CD ellipticity at 288 nm and the UV absorbance at 295 nm vs. time. The folding of the structure results in characteristic hyperchromicity at these wavelengths, both of which are used regularly to study the i-motif. Under the experimental conditions used here the folding kinetics are considerably slower than those observed by Zhou et al. Their work did not explicitly state the concentration of DNA used, only that concentrated aliquots were diluted into buffer pre-incubated at low temperature. In our hands, we were able to, as much as possible without the exact details, replicate their experiments and observed more comparable folding kinetics (Supplementary Figure 3). However, we found using annealed DNA, at a lower concentration, resulted in very different folding kinetics.

It was decided that for this work, a thermal melt and slow anneal should be performed first to reduce variability and ensure that what was being observed was not an artifact of the dilution or sudden change in temperature. A sample was equilibrated at 4°C for 3 h in the spectropolarimeter and its CD and UV signals measured over time; an analogous sample was treated in the same manner, except the equilibration period was extended to 6 h. It can be seen from Supplementary Figure 2 that both samples folded into i-motif over time, identified using CD signals which are characteristic for i-motif: a large positive signal at ~288 nm and a negative signal at ~255 nm. Further samples were stored at 4°C in a refrigerator for up to a week (168 h) and comparison of the CD spectra of the hTeloC samples held at low temperature from 3 to 168 h did not show any noticeable difference; at all of these times the samples of hTeloC at neutral pH folded into i-motif in response to equilibration at low temperature (Supplementary Figure 4). The adoption of i-motif structure under neutral conditions was further confirmed by the hyperchromicity observed over time in both the ellipticity at 288 nm and the absorbance at 295 nm, monitored for the samples equilibrated *in situ*. Under the experimental conditions used here, when compared to the conditions used by Zhou et al. there was a marked decrease in the rate of i-motif folding; with the slow folding process reaching a steady-state equilibrium close to the 3 h mark (Supplementary Figure 5) as opposed to the rapid 700 s observed when concentrated DNA is added to pre-cooled buffer. This dramatic difference in the kinetics of the folding process under these different experimental conditions has implications for the handling of i-motif samples during experimentation and for i-motif-based nanotechnological applications. While this difference was interesting it did not explain the observation from our previous experiments (Figure 1B) where the hTeloC sequence at pH 7 appeared to be folded into i-motif at room temperature. This was hypothesized to possibly be caused by a change in the thermodynamics or kinetics of the system as a result of storage at low temperature. Consequently, samples were once again held at 4°C for different periods of time, equilibrated at 20°C for 30 min, and then melted in the spectropolarimeter. It was found that extended storage at low temperature did not alter the melting temperature of the samples kept at 4°C for increasing lengths of time, with the melting temperatures of all samples being ~28°C (Supplementary Figure 6). This is the first instance of the i-motif formed by the human telomeric sequence existing at such high pH (>7) and at ambient temperature; the cause of this however was not yet understood.

**Figure 2:**
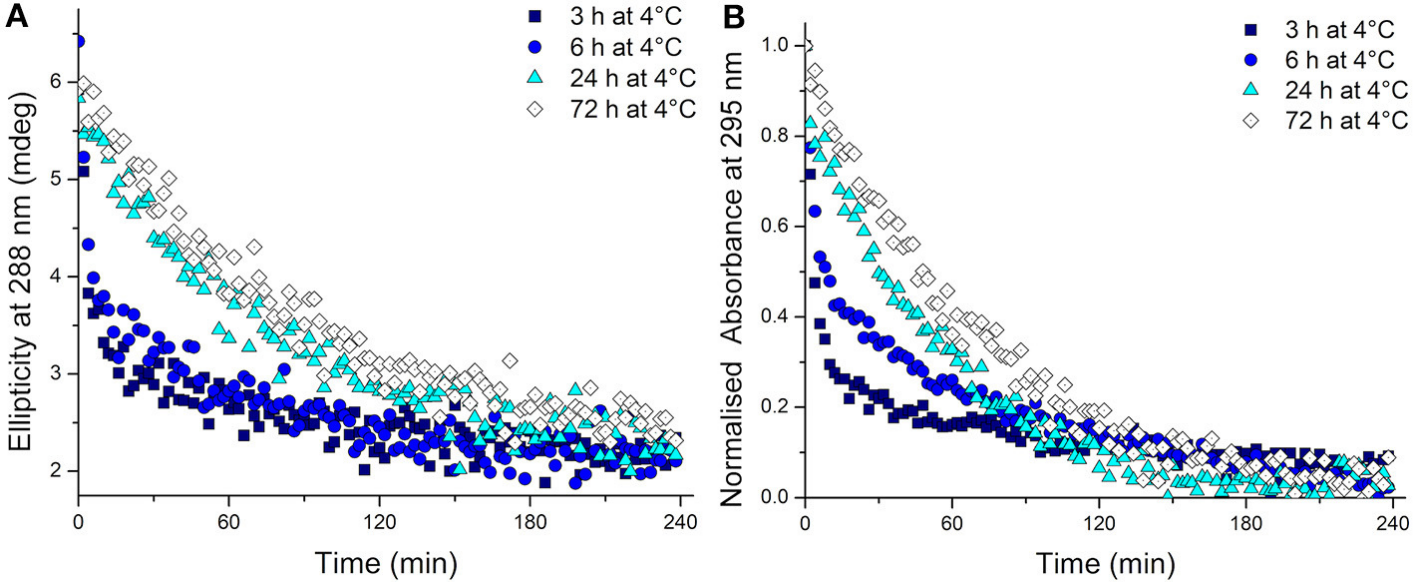
**(A)** Ellipticity at 288 nm and **(B)** normalized absorbance at 295 nm of 10 μM hTeloC in 10 mM sodium cacodylate buffer pH 7.11 measured every 2 min after storage at 4°C for the indicated time.

To test whether the kinetics of this system were affected by extended time at low temperature after equilibration at 4°C, the temperature was changed to 20°C. The CD spectra were measured, this time to monitor the unfolding process, and over time all the samples unfolded as expected (Supplementary Figure 7). Even from these CD spectra, a clear difference is visible in the rate at which the structure unfolds. A more rapid disappearance of the signature i-motif spectra was observed in the samples kept at 4°C for shorter periods of time. Using the ellipticity data at 288 nm and the absorbance at 295 nm and plotting those against time this difference was able to be quantified (Figure 2). It transpired that an increase in equilibration time at 4°C had a dramatic and unexpected effect on the kinetics of the unfolding process. Samples equilibrated at 4°C for 3, 6, 24, and 72 h displayed strikingly different unfolding kinetics; extended time equilibrating at low temperature led to a decrease in the rate of i-motif unfolding at 20°C (Figure 2). These data could be fitted to determine the half-life of the folded structure after different equilibration times (Table 1). From these results it can be seen clearly that extended storage at low temperature leads to a change in the unfolding kinetics where the i-motif unfolds more slowly.

**Table 1:** Half-life values calculated from fitting ellipticity at 288 nm (CD) and normalized absorbance at 295 nm (UV) to one-phase exponential decay functions of 10 μM hTeloC in 10 mM sodium cacodylate buffer pH 7.11 at 20°C after equilibration at 4°C for 3, 6, 24, and 72 h.

**Time at 4°C (h)**	**CD**	**UV**
	**Half-life (min)**	
3	16.4 ± 1.7	8.0 ± 0.6
6	37.2 ± 3.3	49.4 ± 3.4
24	74.8 ± 3.8	53.3 ± 1.4
72	82.8 ± 4.1	68.8 ± 2

In trying to determine the underlying mechanisms that could explain these results it was considered that they were likely caused by some form of structural reconfiguration of the hTeloC i-motif. From the literature it is known that the i-motif is a dynamic structure and it was proposed that in this scenario hTeloC could be adopting a more thermodynamically stable form by (a) changing from an intramolecular i-motif to an intermolecular form or (b) changing between the 3′E and 5′E conformations. The fact that the melting temperature was unaffected by length of time spent at 4°C indicates that the former explanation is unlikely. The melting temperature of an intermolecular i-motif structure is concentration dependent (Mergny et al., 1995) and although all of the samples used in this work had a DNA concentration of 10 μM, a slow, low-temperature induced, formation of i-motif would result in there being different concentrations of the intermolecular structure at the different time points; and consequently a difference in the melting temperatures, which we do not observe. Furthermore, the latter explanation mirrors exactly what was described by Lieblein et al. when studying the folding of their variant of the human telomeric i-motif sequence 5′-[(CCC-TAA)_3_CCC]-3′ (which differs from the hTeloC sequence used here only by the absence of three flanking bases). Their study found that in response to a drop in pH from 9 to 6 their sequence formed the less stable 3′E conformation more rapidly than the more stable 5′E conformation (Lieblein et al., 2012). Therefore, it seems from the data we have gathered that an analogous conformational change occurs at low temperature with hTeloC at slightly alkaline pH: it more rapidly forms a kinetically favored, but less stable, conformation and this converts slowly to a more thermodynamically stable conformation over time.

**Figure 3:**
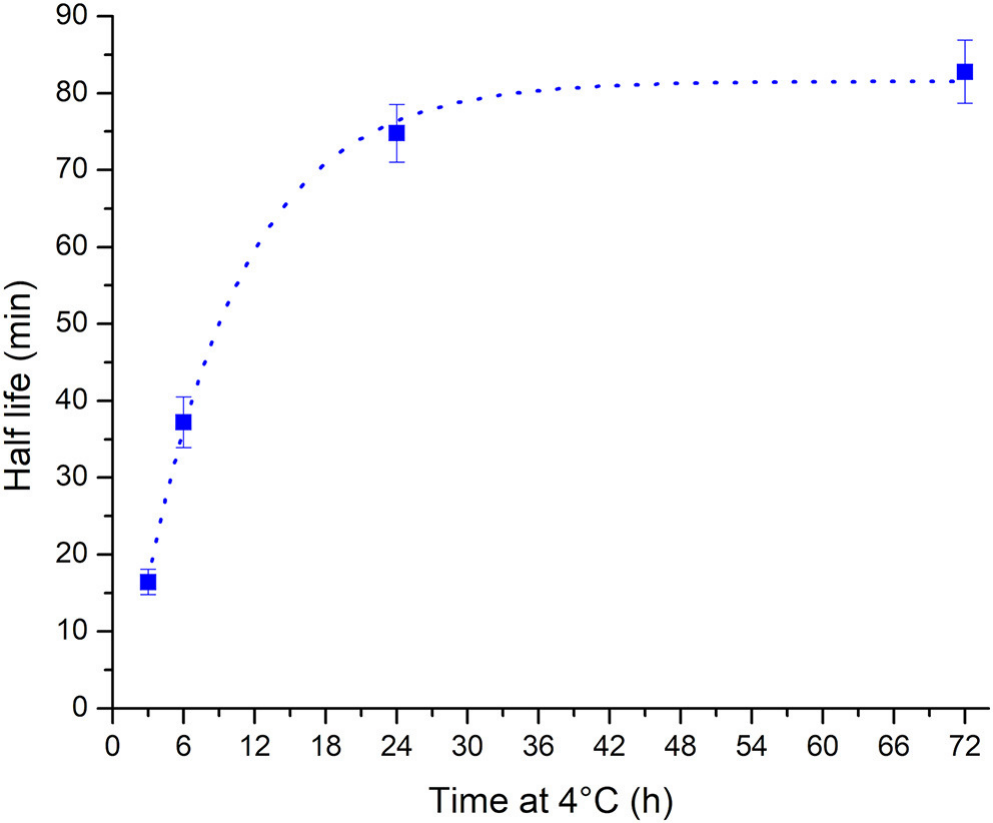
Plot of half-life vs. storage time at 4°C showing that the half-life increases with increasing length of time at low temperature. The relationship is well fitted to a one phase exponential growth function (dotted line).

The observed change in the unfolding kinetics explains the initial unintended observation of folded i-motif at pH 7 at room temperature after storage of samples in the refrigerator between measurements. While the samples were taken out of the refrigerator so that they could return to room temperature prior to measurement, the only consideration cannot simply be the sample's temperature but the extension in the unfolding half-life must also be considered. The calculated half-life values vs. the length of time spent at 4°C are well fitted (*R*^2^ = 0.997) to a one-phase exponential growth function (Figure 3). Using this method the half-life of the i-motif at a given temperature after cold storage for different lengths of time can be calculated. The cautionary tale of this surprisingly slow change in the kinetics of the i-motif's unfolding has led to the discovery of a phenomenon that adds a further avenue in the design of i-motif based nanotechnologies. For example, slow unfolding kinetics could be utilized in the release of a drug over a period of hours using these conditions. In addition to having systems which have their response tuned to the myriad combinations of changes in pH, cations or temperature; this introduces the ability to control the rate of unfolding of i-motif sensors and systems on the order of minutes or hours as opposed to the rapid transitions seen in response to changes in pH or temperature and without having to change its environment. This finding also lends credence to the i-motif playing a role in the control of biological processes. This sequence from the human telomeric region has not been known previously to be able to exist in the folded i-motif state above neutral pH and at room temperature. Knowing now the dramatic changes possible in the thermodynamic and kinetic stability of the structure it is not unreasonable to imagine how physiological conditions of molecular crowding, superhelical stress, interactions with proteins, or many others, could give rise to stable i-motifs *in vivo*.

We have found that the i-motif forming sequence from the human telomeric region not only folds into i-motif above neutral pH at low temperature. Moreover, this quadruplex structure can persist in the same conditions of neutral pH and ambient temperature in which, once at thermodynamic equilibrium, it exists predominantly as a random coil. The i-motif unfolding kinetics were found to be dependent on the time allowed for annealing and equilibration, and crucially the temperature at which samples are maintained. This research demonstrates the importance of consistency when annealing and storing i-motif samples, and highlights the dynamic nature of the quadruplex. Our findings have implications not only for work with i-motif DNA structures, but also in the uses and applications of i-motifs in nanotechnological devices.

## Data Availability Statement

The raw data supporting the conclusions of this article will be made available by the authors, without undue reservation, to any qualified researcher.

## Author Contributions

MA and ZW conceived and designed the experiments and wrote the paper. MA performed the experiments and analyzed the results. Both authors verified the data, contributed to the manuscript, and approved the final version.

### Conflict of Interest

The authors declare that the research was conducted in the absence of any commercial or financial relationships that could be construed as a potential conflict of interest.
