# Supplementary material for: Tricky Topology: Persistence of Folded Human Telomeric i-Motif DNA at Ambient Temperature and Neutral pH
Source: Front Chem. 2020 Jan 31;8:40. doi: 10.3389/fchem.2020.00040 (PMC7005205; doi:10.3389/fchem.2020.00040)
Supplement: Supplementary file 1 [file Data_Sheet_1.docx]

Supplementary Material

# Supplementary Figures


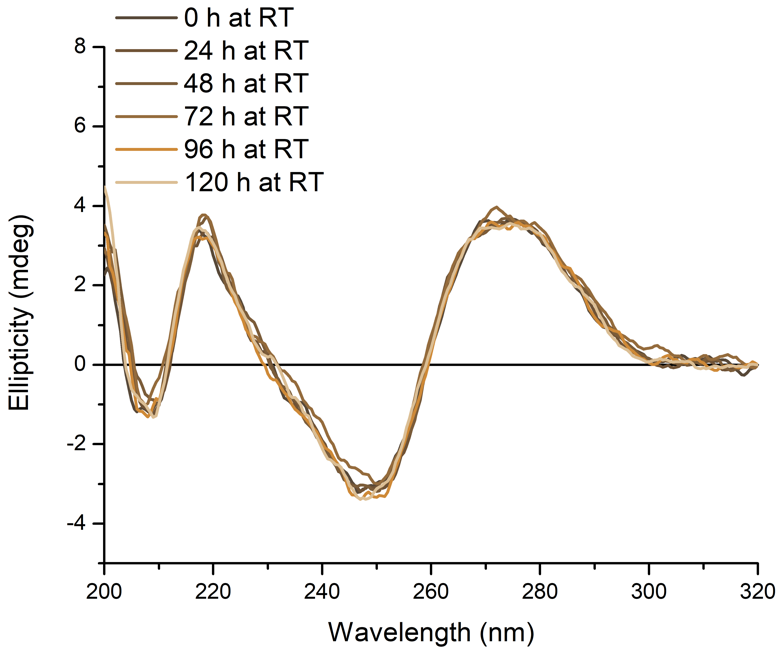


**Supplementary Figure 1.** CD spectra of 10 µM hTeloC in 10 mM sodium cacodylate buffer pH 7.11 after storage at room temperature (~20°C) for the indicated time.


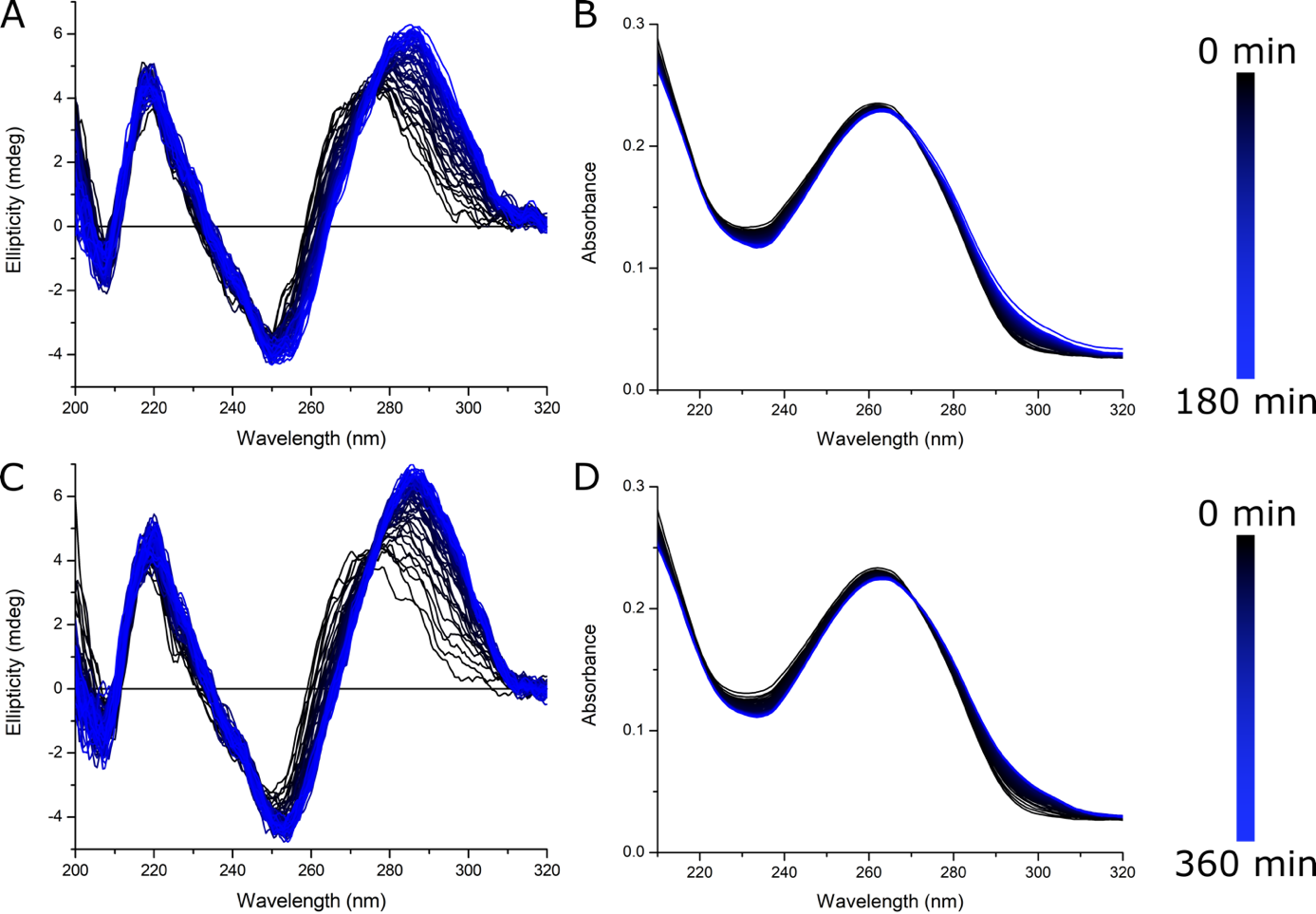


**Supplementary Figure 2.** CD (A,C) and UV (B,D) spectra of 10 µM hTeloC in 10 mM sodium cacodylate buffer pH 7.11. Samples were thermally annealed by holding at 95°C for five minutes and cooled slowly to room temperature overnight. Acquisition of spectra began when the temperature of the sample holder reached 4°C and measured every two minutes for three hours (upper panels) and every five minutes for 6 hours (lower panels).


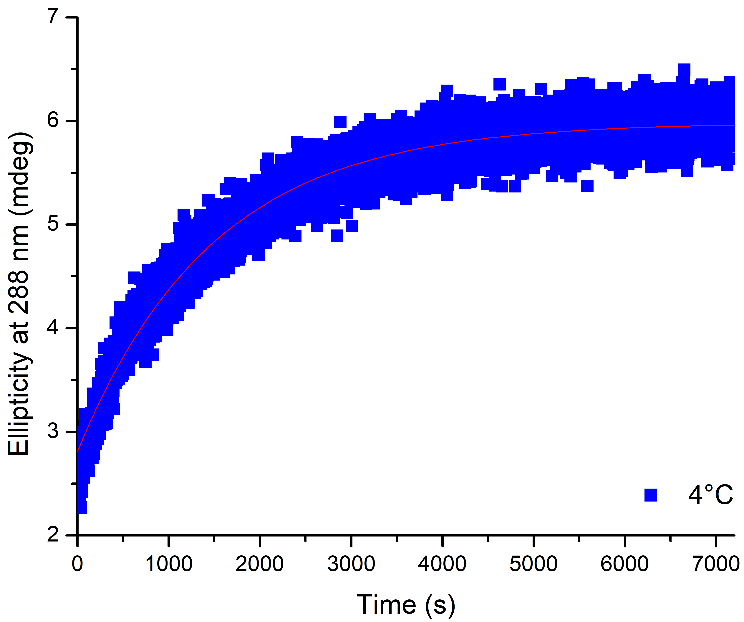


**Supplementary Figure 3.** Ellipticity at 288 nm of 10 µM hTeloC diluted from ~1 mM stock in ultrapure water into 10 mM sodium cacodylate buffer pH 7.0 pre-incubated at 4°C. Sample was diluted, mixed quickly and ellipticity at 288 nm measured at 0.5 s intervals for two hours.


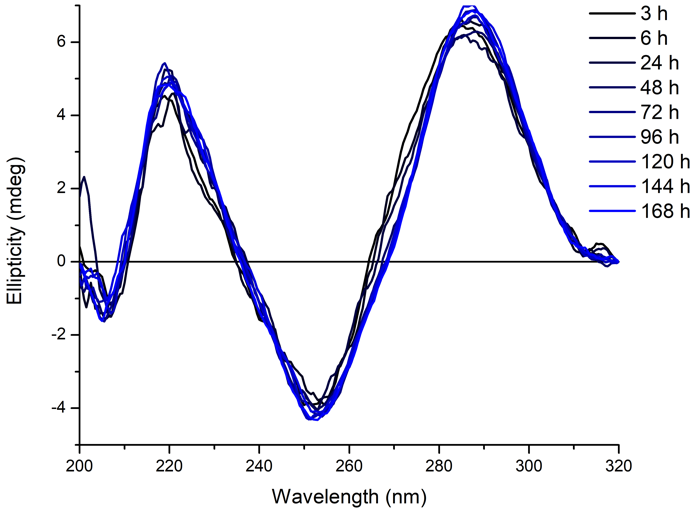


**Supplementary Figure 4.** CD spectra of 10 µM hTeloC in 10 mM sodium cacodylate buffer pH 7.11 after storage at 4°C for the indicated time.


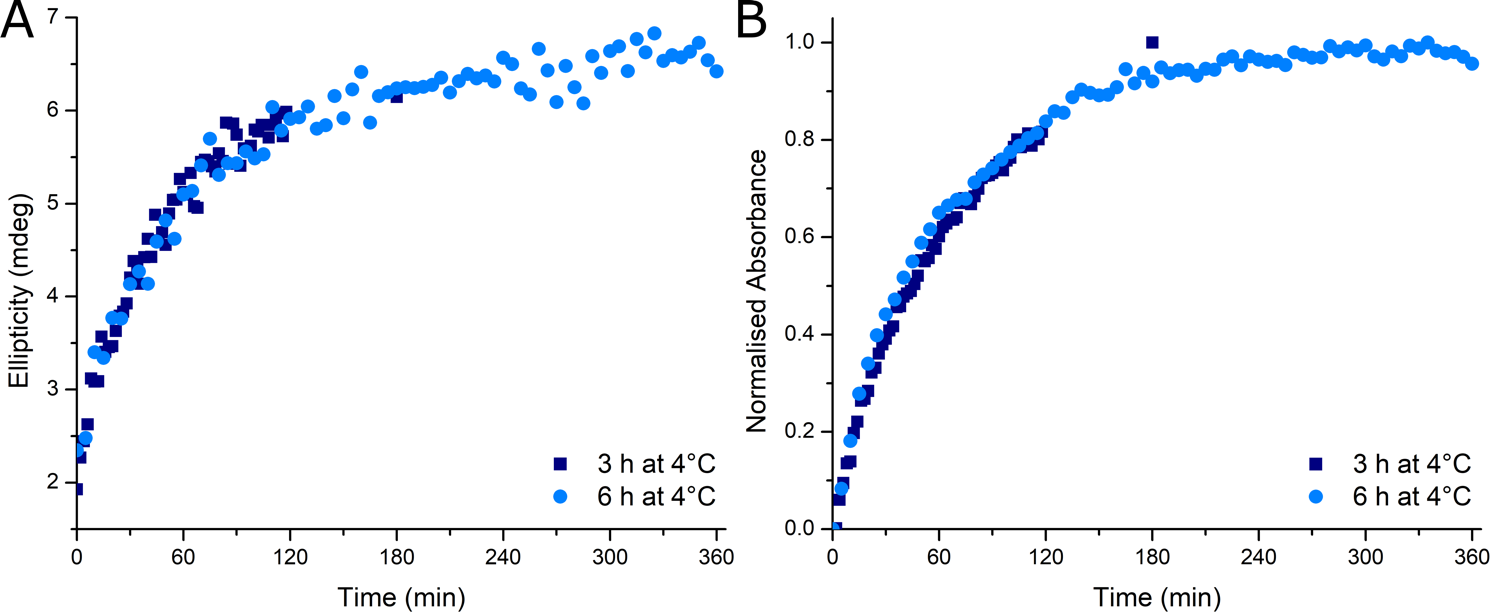


**Supplementary Figure 5.** Ellipticity at 288 nm (A) and normalised absorbance at 295 nm (B) of 10 µM hTeloC in 10 mM sodium cacodylate buffer pH 7.11. Samples were thermally annealed by holding at 95°C for five minutes and cooled slowly to room temperature overnight. Acquisition of spectra began when the temperature of the sample holder reached 4°C and measured every two minutes for three hours (■ – dark blue square) and every five minutes for 6 hours (● – light blue circle).


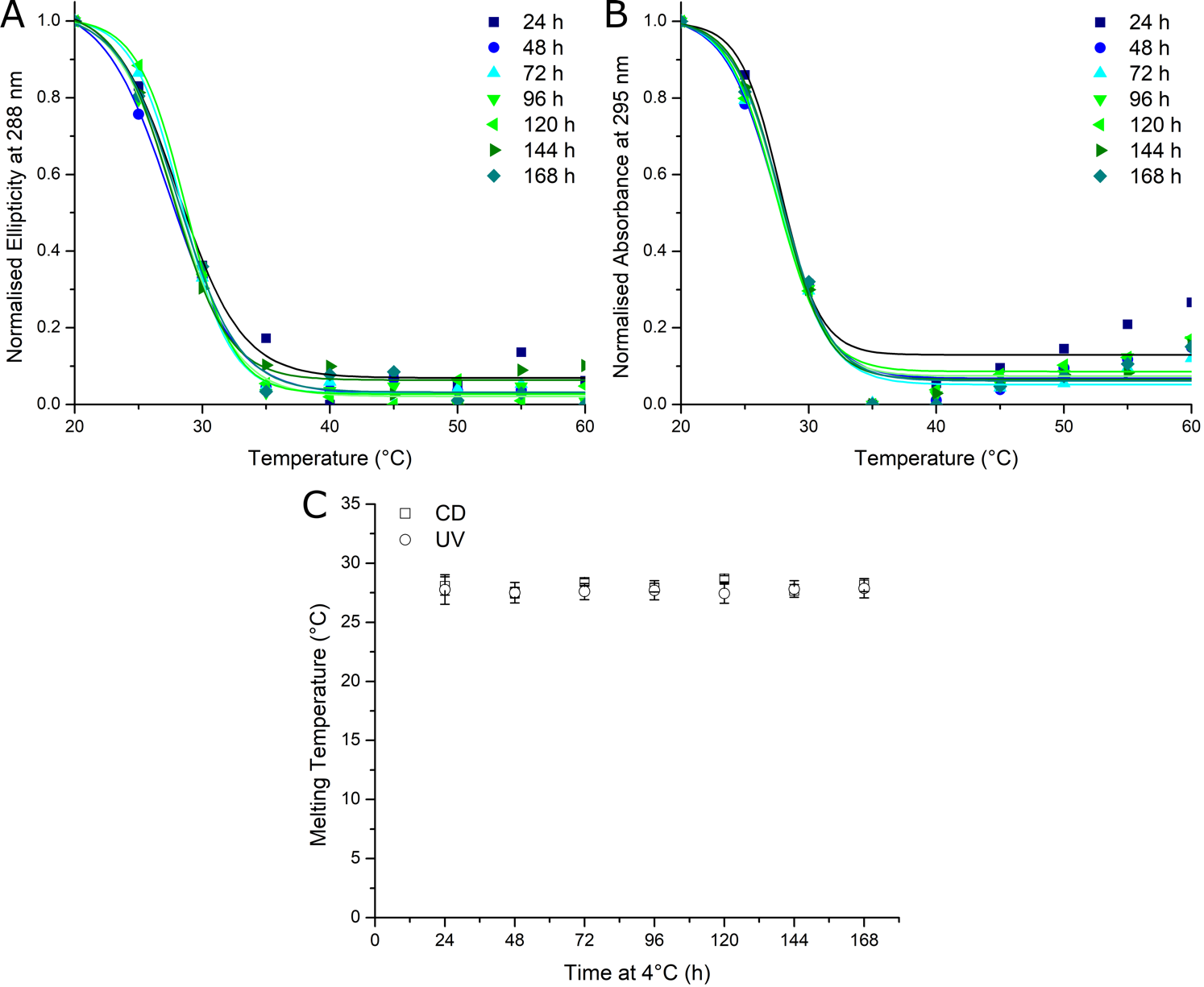


**Supplementary Figure 6.** Normalised ellipticity at 288 nm (A) and normalised absorbance at 295 nm (B) of 10 µM hTeloC in 10 mM sodium cacodylate buffer pH 7.11. Samples were thermally annealed by holding at 95°C for five minutes and cooled slowly to room temperature overnight. They were then stored at 4°C for the indicated time, equilibrated at 20°C for 30 minutes and then melted in the spectropolarimeter. Melting temperature (C) of samples calculated using data from upper panels.


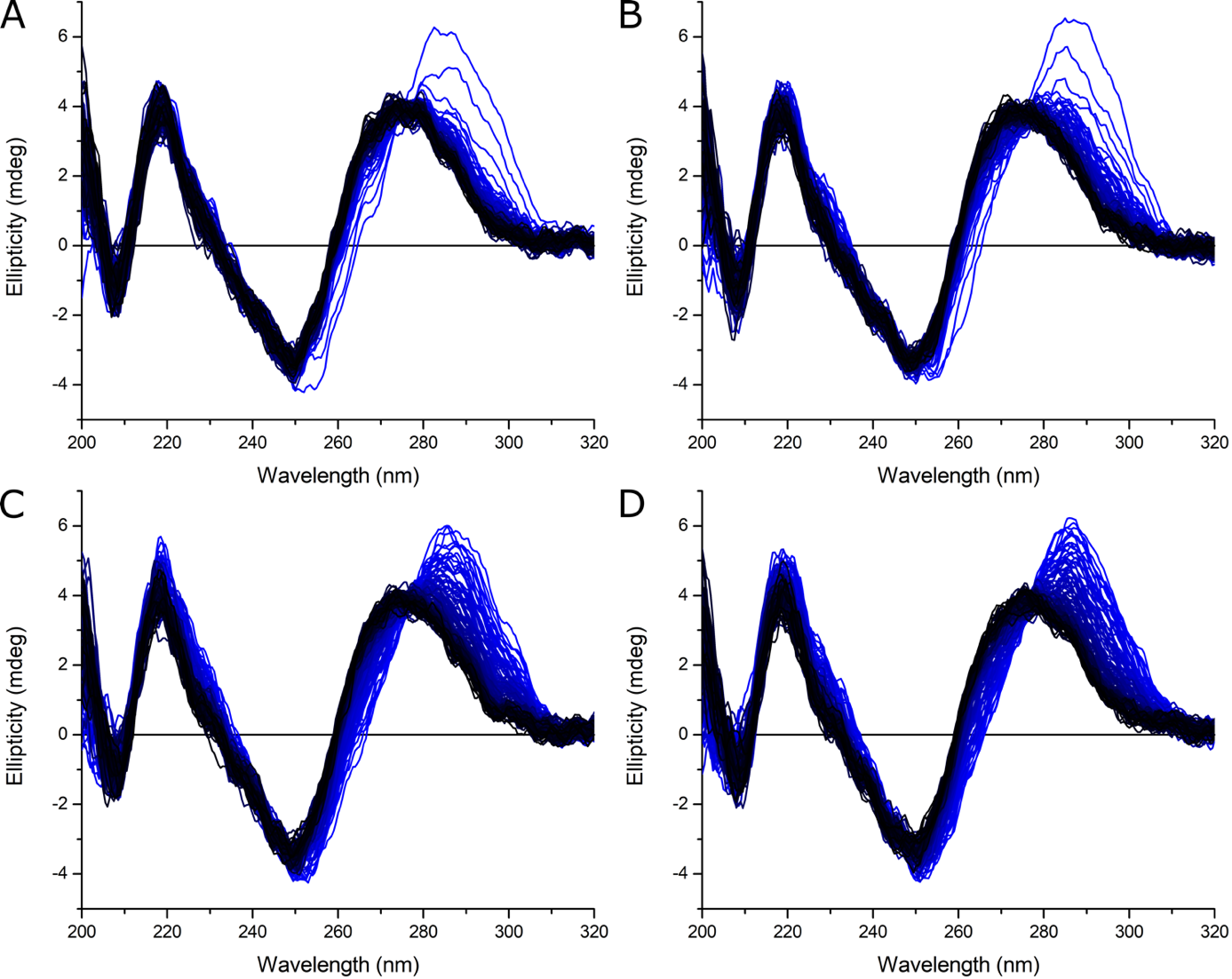


**Supplementary Figure 7** CD (A,C) and UV (B,D) spectra of 10 µM hTeloC in 10 mM sodium cacodylate buffer pH 7.11. Samples were thermally annealed by holding at 95°C for five minutes and cooled slowly to room temperature overnight. They were then held at 4°C for 3 (A), 6 (B), 24 (C) and 72 (D) hours. The temperature was then changed to 20°C and acquisition of spectra began when the temperature of the sample holder reached the target and measured every two minutes for four hours.
